# Dynamic Functional Connectivity Better Predicts Disability Than Structural and Static Functional Connectivity in People With Multiple Sclerosis

**DOI:** 10.3389/fnins.2021.763966

**Published:** 2021-12-13

**Authors:** Ceren Tozlu, Keith Jamison, Susan A. Gauthier, Amy Kuceyeski

**Affiliations:** ^1^Department of Radiology, Weill Cornell Medicine, New York, NY, United States; ^2^Judith Jaffe Multiple Sclerosis Center, Weill Cornell Medicine, New York, NY, United States; ^3^Department of Neurology, Weill Cornell Medical College, New York, NY, United States; ^4^Brain and Mind Research Institute, Weill Cornell Medicine, New York, NY, United States

**Keywords:** multiple sclerosis, structural connectivity, functional connectivity, machine learning, predictive modeling

## Abstract

**Background:** Advanced imaging techniques such as diffusion and functional MRI can be used to identify pathology-related changes to the brain's structural and functional connectivity (SC and FC) networks and mapping of these changes to disability and compensatory mechanisms in people with multiple sclerosis (pwMS). No study to date performed a comparison study to investigate which connectivity type (SC, static or dynamic FC) better distinguishes healthy controls (HC) from pwMS and/or classifies pwMS by disability status.

**Aims:** We aim to compare the performance of SC, static FC, and dynamic FC (dFC) in classifying (a) HC vs. pwMS and (b) pwMS who have no disability vs. with disability. The secondary objective of the study is to identify which brain regions' connectome measures contribute most to the classification tasks.

**Materials and Methods:** One hundred pwMS and 19 HC were included. Expanded Disability Status Scale (EDSS) was used to assess disability, where 67 pwMS who had EDSS<2 were considered as not having disability. Diffusion and resting-state functional MRI were used to compute the SC and FC matrices, respectively. Logistic regression with ridge regularization was performed, where the models included demographics/clinical information and either pairwise entries or regional summaries from one of the following matrices: SC, FC, and dFC. The performance of the models was assessed using the area under the receiver operating curve (AUC).

**Results:** In classifying HC vs. pwMS, the regional SC model significantly outperformed others with a median AUC of 0.89 (*p* <0.05). In classifying pwMS by disability status, the regional dFC and dFC metrics models significantly outperformed others with a median AUC of 0.65 and 0.61 (*p* < 0.05). Regional SC in the dorsal attention, subcortical and cerebellar networks were the most important variables in the HC vs. pwMS classification task. Increased regional dFC in dorsal attention and visual networks and decreased regional dFC in frontoparietal and cerebellar networks in certain dFC states was associated with being in the group of pwMS with evidence of disability.

**Discussion:** Damage to SCs is a hallmark of MS and, unsurprisingly, the most accurate connectomic measure in classifying patients and controls. On the other hand, dynamic FC metrics were most important for determining disability level in pwMS, and could represent functional compensation in response to white matter pathology in pwMS.

## 1. Introduction

Multiple Sclerosis (MS) is a chronic disease characterized by inflammatory and demyelinating lesions within the central nervous system (Weinshenker et al., 1991). One key observation is that the disease burden in the brain, as measured with conventional imaging, is not always proportional to an individual's disability. Individuals can have identical lesion volume and very different levels of disability (Barkhof, 2002), making prognostication in this disease challenging. Advanced neuroimaging techniques may enable us to better understand the neuropathological mechanisms of MS, how they cause disability in MS and how the brain may compensate for this pathology. Brain connectivity network analysis, or connectomics, provides a promising tool with which to map the effect of MS-related pathology and to potentially capture reorganization mechanisms in response to pathology. Inflammation, demyelination, and axonal loss in people with MS (pwMS) disrupts the brain's structural connectome (SC), which may contribute to some of the changes observed in the brain's regional activation patterns, or functional connectome (FC) (Rocca et al., 2010, 2012, 2018; Roosendaal et al., 2010; Tona et al., 2014; Schoonheim et al., 2015).

Previous studies have used SC and FC separately or together to identify differences between pwMS and healthy controls (HC), to compare different clinical categories of MS, and to classify pwMS by disability or cognitive impairment level (Richiardi et al., 2012; Kocevar et al., 2016; Zhong et al., 2017; Saccà et al., 2018; Zurita et al., 2018; Has Silemek et al., 2020). It has been shown that alterations in the SC and/or FC in particular networks are associated with motor and cognitive impairment in pwMS (Faivre et al., 2012; Rocca et al., 2012; Basile et al., 2014; Filippi et al., 2015; Kuceyeski et al., 2015, 2018). SC damage may cause an upregulation of FC in specific networks as a compensatory mechanism in the early stages of MS, which then wanes in the later stages of the disease. In particular, the Expanded Disability Status Scale (EDSS) threshold of 3 was previously identified as the cut-off for functional reorganization and adaptation in MS (Hawellek et al., 2011; Faivre et al., 2012; Tommasin et al., 2018). Two individuals with the same pattern of SC damage may have different disability levels depending on where they are in the trajectory of compensatory FC, thus, FC may be more informative of disability than SC in this case.

An individual's FC is usually obtained by correlating regional Blood Oxygenation Level Dependent (BOLD) signals acquired over the entire functional MRI (fMRI) scan; however, this “static” FC derivation does not consider the fluctuations in the brain network topology that can occur over time (Biswal et al., 1995; Damaraju et al., 2014). Dynamic FC (dFC) approaches allow assessment of the varying topology of FC over time by using sliding windows to assess dynamic FCs (Allen et al., 2014). There is increased interest in using dFC to investigate pathological mechanisms in psychiatric disorders, and stroke (Damaraju et al., 2014; Rashid et al., 2016; Sambataro et al., 2017; Mennigen et al., 2018; Bonkhoff et al., 2020, 2021). In MS, recent studies have used dFC to (1) compare clinically isolated syndrome (CIS) patients to HC (Rocca et al., 2019), (2) analyze relationships with information processing speed in relapsing-remitting (RR) pwMS (van Geest et al., 2018), and (3) classify cognitively impaired vs. preserved pwMS (d'Ambrosio et al., 2019; Eijlers et al., 2019). However, no study to date has performed a rigorous analysis of the relative contributions of multi-modal imaging data including SC, static FC, and dynamic FC in classifying HC vs. pwMS and/or pwMS by disability status. Understanding the relative contributions of the various modalities may provide insight into disability-relevant disease or compensatory mechanisms.

Therefore, the principal aim of the present study was to compare the performance of either pairwise or regional SC, FC, and dFC metrics in classifying (1) HC vs. pwMS and (2) pwMS who had no disability vs. those who had evidence of disability. The secondary aim was to identify the most important pairwise and regional connections as well as dFC metrics for the classification tasks. Diffusion and resting-state functional MRI were used to compute the SC and FC matrices, respectively. We hypothesized that models including SC could best distinguish HC from pwMS, as white matter lesions impacting SC networks are a hallmark of the disease. Furthermore, we hypothesized that models containing FC and/or dFC would best distinguish disability levels in pwMS as this modality likely is sensitive to functional compensation mechanisms that may underlie disability in MS. Overall, our goal is to better understand mechanisms of pathology and resilience in MS, knowledge which could be used to improve the accuracy of prognoses and even develop novel therapies to reduce disability.

## 2. Materials and Methods

### 2.1. Subjects

One hundred pwMS (median age: 45.5 [36.7, 56.0], 66% females) with a diagnosis of Clinically Isolated Syndrome (CIS)/MS based on the 2010 McDonald criteria (Polman et al., 2011) [CIS = 7, relapsing-remitting MS (RRMS) = 88, primary progressive MS (PPMS) and secondary progressive MS (SPMS) = 5] and 19 HC (median age: 45 [35, 49], 55% females) were included in our study. Seven people with CIS were included in the group of pwMS as they are likely an early form of MS, they were all in the no disability group (EDSS < 2). MRIs and demographic data were collected (age, sex, and race for both HC and pwMS, clinical phenotype, and disease duration for pwMS). All subjects with SC and FC networks available were included in our study and a power analysis was not performed prior to the statistical analysis. Participants were excluded if they had contraindications to MRI or had ever been diagnosed or were currently on medication for a neurological or psychological disorder (other than a diagnosis of or medication for treatment of MS for the pwMS group, of course). The spinal cord lesion category was estimated from the patient's clinical radiology report, with 0 indicating those with no spinal lesions, 1 indicating those with one spinal lesion or 2 indicating those with more than one spinal lesion. The EDSS is the most frequently used disability scale in MS and captures mostly the motor functioning. EDSS ranges from 0 to 10 with 0.5-unit increments, where 0 indicates no disability and the increase in EDSS indicates higher level of disability. EDSS was used to quantify disability in our study, where an EDSS of 2 was used as a threshold to categorize disablity status: no disability (EDSS < 2) vs. evidence of disability (EDSS ≥ 2). This group division was based on EDSS values of 0–1.5 representing some abnormal signs in neurological examination but no functional impairment is appreciated. This was a cross-sectional study, and the MRIs and demographics/clinical data were collected the same year. All studies were approved by an ethical standards committee on human experimentation and written informed consent was obtained from all patients.

### 2.2. Image Acquisition, Processing, and Connectome Extraction

MRI data were acquired on a 3T Siemens Skyra scanner (Siemens, Erlangen, Germany) with a 20-channel head-neck coil and a 32-channel spine-array coil. Anatomical MRI (T1/T2/T2-FLAIR, 1 mm3 iso-voxel), resting-state fMRI (6 min, TR = 2.3 s, 3.75 × 3.75 × 4 mm voxels) and diffusion MRI (55 directions HARDI, *b* = 800, 1.8 × 1.8 × 2.5 mm voxels) acquisitions were performed. Sagittal STIR images were acquired for identification of spinal lesions (TR = 3.5 s, TI = 220 ms, TE = 45 ms, in-plane resolution 0.43 mm, FOV = 22 mm, slice thickness 3 mm). Multi-echo 2D GRE fieldmaps were collected for use with both fMRI and diffusion MRI (0.75 × 0.75 × 2 mm voxels, TE1 = 6.69 ms, △TE = 4.06 ms, number of TEs = 6). The white and gray matter surfaces were checked for each subject on Freesurfer and hand-edited with control points and reconstruction editing if necessary.

White matter (WM) and gray matter (GM) were segmented and GM further parcellated into 86 regions of interest (68 cortical and 18 subcortex/cerebellum) using FreeSurfer (Fischl and Dale, 2000). As described elsewhere (Kuceyeski et al., 2016), fMRI preprocessing included simultaneous nuisance regression and removal of WM and cerebrospinal fluid (CSF) effects (Hallquist et al., 2013), followed by band-pass filtering (0.008–0.09 Hz) using the CONN v18b toolbox (Whitfield-Gabrieli and Nieto-Castanon, 2012) and SPM12 in Matlab. Nuisance regressors included 24 motion parameters (6 rotation and translation, temporal derivatives, and squared version of each) and the top 5 eigenvectors from eroded masks of both WM and CSF. The mean fMRI signal over all voxels in a region was calculated and the mean regional time series correlated (Pearson's correlation) between every pair of regions to obtain pairwise FC matrices. Regional FC node strengths were calculated by taking the sum of the columns in the FC matrix after removing the negative entries.

Diffusion MRI was interpolated to isotropic 1.8 mm voxels, and then corrected for eddy current, motion, and EPI-distortion with the eddy command from FSL 5.0.11 (Andersson and Sotiropoulos, 2016) using the outlier detection and replacement option (Andersson et al., 2016). MRtrix3Tissue (https://3Tissue.github.io), a fork of MRtrix3 (Tournier et al., 2019) was used to estimate a voxel-wise single-shell, 3-tissue constrained spherical deconvolution model (SS3T-CSD) and then compute whole-brain tractography for each subject. The SC matrix was constructed by taking the sum of the SIFT2 weights of streamlines connecting pairs of regions and then dividing by the sum of the two regions' volumes. In addition to the pairwise SC measures, regional SC node strength was quantified by taking the sum of each of the columns in the SC matrix.

### 2.3. Dynamic FC Analysis

Dynamic FC matrices were calculated using a tapered, sliding window approach in the GIFT toolbox (http://mialab.mrn.org/software/gift) (Allen et al., 2014; Damaraju et al., 2014). The BOLD time series that were extracted from 86 regions of FreeSurfer atlas (same atlas used for static FC and SC analysis) were used as an input to the GIFT toolbox. As suggested by Allen et al. (2014) and previous studies (Bonkhoff et al., 2020, 2021), dFC between two regional time courses was computed using a sliding window approach with a window size of 22 TR (50.6 s) in steps of 1 TR (2.3 s). A rectangular window of 22 time points convolved with a Gaussian of 3 TR (6.9 s) was used for tapering along the edges, resulting in 153 tapered time windows per subject. Once the dFC matrices were calculated, k-means clustering was applied to all dFC matrices to identify clusters of reoccurring dFC states. The elbow criterion, i.e. the ratio of within-cluster to between-cluster Manhattan (L1) distances, was used to identify the optimal number of clusters. The following metrics were extracted from the dFC analysis: (1) mean dwell time in each state (= how long the individual remains in a state once they transition to it), (2) transition probability from one dFC state to another between two consecutive time points, and (3) the number of overall state transitions in the scan. We extracted individuals' cluster centroids for each of the dFC states as the mean dFC of each dFC assigned to a particular cluster. Further, we took the node strength of the individuals' cluster centroids as a “regional dFC” measure (after removing negative entries in the dFC). Network-level interpretations were enabled by assigning each of the 86 gray matter regions to one of the 7 Yeo functional networks, plus a subcortical and a cerebellum network (Yeo et al., 2011).

### 2.4. Mass Univariate Analysis

First, demographics and clinical variables were tested for differences between the groups [(1) HC vs. pwMS and (2) pwMS who had no disability (EDSS < 2) vs. evidence of disability (EDSS ≥ 2)] using Chi-squared test for qualitative variables, Wilcoxon rank-sum test for quantitative variables. As a *post-hoc* analysis, ANOVA was applied to compare the age, EDSS, and phenotypes between two MS disability groups A *t*-test was used to compare pairwise entries in static FC and dFC summary metrics, while Wilcoxon rank-sum test was performed to compare SC values between groups. Only pairwise connections in the SC that were non-zero in more than half of the controls were tested for differences between groups to minimize the effect of false positives in the tractography results. Differences were considered significant when *p* < 0.05 after Benjamini-Hochberg (BH) correction for multiple comparisons (Benjamini and Hochberg, 1995). All statistical analyses and graphs were performed using R (www.r-project.org), version 3.4.4 and Matlab (https://www.mathworks.com/) version R2020a.

### 2.5. Classification Analysis

**Figure 1:**
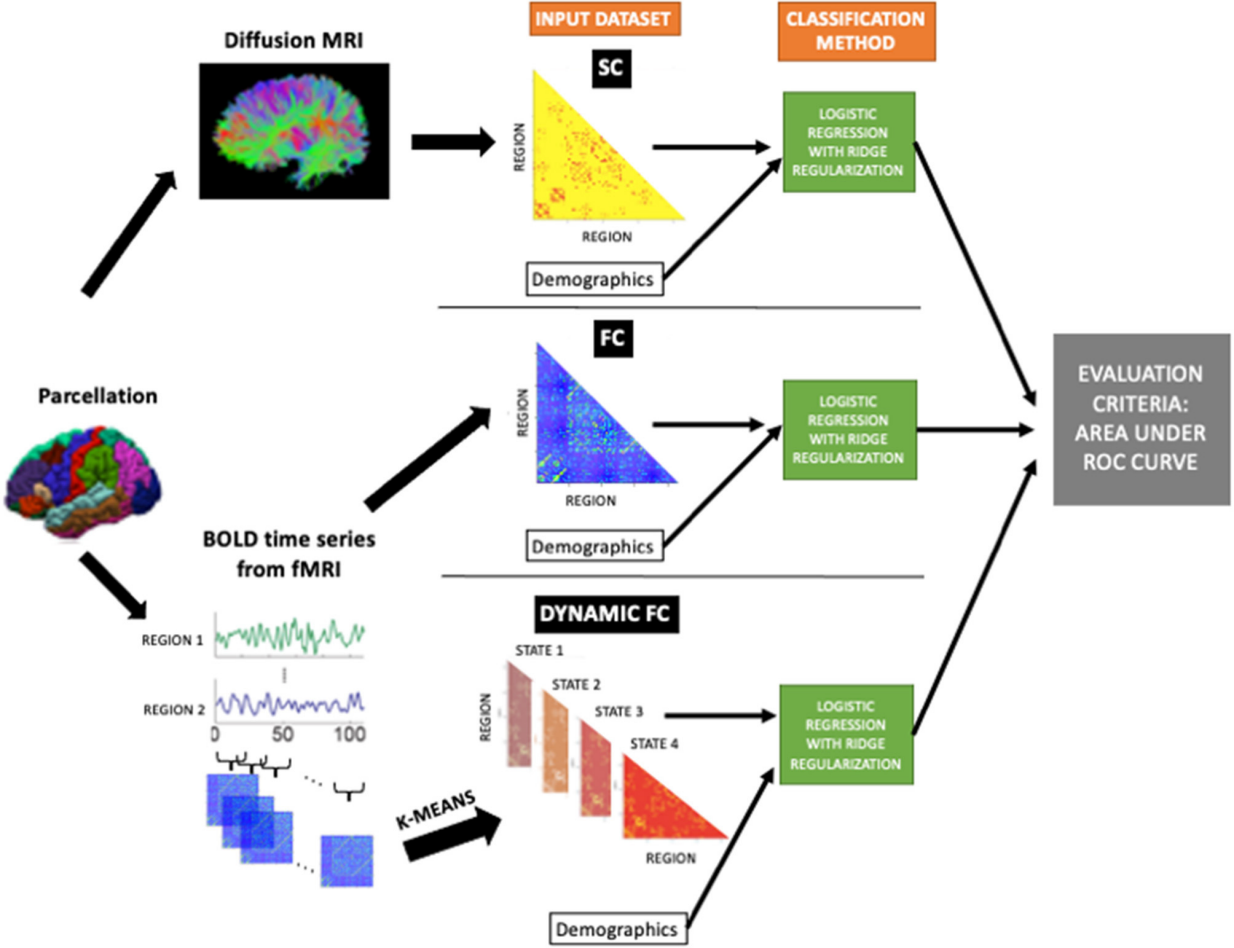
The workflow of the study. Structural connectomes (SC), static functional connectomes (FC), and dynamic functional connectome (dFC) metrics were used as input, in addition to the demographics/clinical variables, to the classification model (logistic regression with ridge regularization technique). The classification performance of each model was assessed using the area under the ROC curve (AUC).

Logistic regression with ridge regularization was used to classify (1) HC vs. pwMS and (2) pwMS who had no disability (EDSS < 2) vs. evidence of disability (EDSS ≥ 2). The classification models used demographics/clinical information (sex, age, race, disease duration, clinical phenotype, and spinal lesion burden category) and one of the pairwise or regional imaging data: SC, FC, dFC. For the HC vs. pwMS classification, only sex, age, and race were used as demographics/clinical variables. Figure 1 shows the overall workflow of the study including the input datasets (SC, FC, and dFC in addition to the demographics and clinical variables) that were used in the various models.

The models were trained with outer and inner loops of k-fold cross-validation (*k* = 5) to optimize the hyperparameters and test model performance. The folds for both inner and outer loops were stratified to ensure that each fold contained the same proportion of subjects in the two classes as the original dataset. The inner loop (repeated over 5 different partitions of the training dataset only) optimized the set of hyperparameters that maximized validation AUC. A model was then fitted using the entire training dataset and those optimal hyperparameters, which was then assessed on the hold-out test set from the outer loop. The outer loop was repeated for 100 different random partitions of the data (see Supplementary Figure 5). The median of AUC (over all 5-folds × 100 iterations = 500 test sets) were calculated to assess the performance of the models. In addition to the AUC results; sensitivity, specificity, balanced accuracy (average of sensitivity and specificity), and F1 scores ($\frac{\text{TP}}{\text{TP}+1/2\times \left(\text{FP + FN}\right)}$) where TP is the number of true positives, FP is the number of false positives, and FN is the number of false negatives are also provided to compare the results of the current study with previous findings. The classification performance of different models were compared using a permutation test (David, 2008). Differences were considered significant when *p* < 0.05 after BH correction for multiple comparisons (Benjamini and Hochberg, 1995).

When the data contains class imbalance, models may tend to favor the majority class. Due to the class imbalance in our data (19 HC vs. 100 pwMS and 67 pwMS with no disability vs. 33 pwMS with evidence of disability), the over-sampling approach Synthetic Majority Over-sampling Technique (SMOTE) (Chawla et al., 2002) was used to obtain a balanced training dataset during the cross-validation. SMOTE compensates for imbalanced classes by creating synthetic examples using nearest neighbor information and has been shown to be among the most robust and accurate methods with which to control for imbalanced data (Santos et al., 2018).

We considered feature weights in the ridge classification method to be the average parameter coefficient over all 500 models (100 partitions of the data × 5-folds). Feature weights of the pairwise connections were represented using a circle plot and summarized at a network level, while feature weights of the regional connections were illustrated via glass brains and summarized at a network level via circle plots. Important connectomic features were identified as those that had both large group differences (via the mass univariate statistical tests) and large feature weights from the ridge classifier (Tian et al., 2021).

## 3. Results

### 3.1. Patient Characteristics

Table 1 shows the subjects' demographic and clinical information including sex, age, disease duration, EDSS, and spinal cord lesion number. Age and sex were not significantly different between HC and pwMS (corrected *p* > 0.05 for both). Unsurprisingly, pwMS who had no disability were younger (corrected *p* < 0.05) and had a trend toward shorter disease duration (corrected *p* = 0.06) compared to pwMS who had evidence of disability. The phenotype and disability groups were not independent (corrected *p* < 0.05), where the pwMS who had CIS phenotype were included in the no disability group and those who had progressive disease were included in the evidence of disability group. The *F*-values obtained with the ANOVA for the age was 16.65 (*p* < 9.17e-05), for the EDSS 143.3 (*p* < 2e-16), and for the phenotypes 7.67 (*p* < 0.0008). However, the two disability groups did not have a significant difference in sex ratio, number of spinal cord lesions, and brain lesion volume (corrected *p* > 0.05 for all).

**Table 1:** Subject demographics and clinical information.

**Variable**	**HC (*n* = 19)**	**pwMS (*n* = 100)**	***p*-value**	**pwMS: no disability**	**pwMS: evidence of**	***p*-value**
				**(*n* = 67)**	**disability (*n* = 33)**	
Age	45 [35.55, 49.50]	45.50 [36.75, 56]	0.84	40 [35, 50]	56 [46, 58]	0.0001
Female (%)	11 (55)	66 (66)	0.49	46 (69%)	20 (61%)	0.56
Disease duration	-	11 [7,16]	-	10 [7,15]	13 [9,17]	0.06
EDSS	-	1 [0, 2]	-	0 [0, 1]	2 [2, 3]	<2.2e-16
Number of spinal cord lesions	-	1 [0,3]	-	1 [0,3]	2 [0,3]	0.46
Phenotype	-	7 CIS, 88 RRMS, 5 Progressive MS	-	7 CIS, 60 RRMS	28 RRMS, 5 Progressive MS	<2.2e-16
Lesion volume (mm^3^)	-	2,065 [717, 4,779]	-	1,995 [734, 4,200]	2,482 [453, 7,788]	0.49

*Values are presented as median [1st, 3rd quantile] for the continuous variables as the number/percent for sex and phenotype. The HC vs. pwMS as well as two disability groups of pwMS were tested for differences; p-values shown are corrected for multiple comparisons*.

### 3.2. Dynamic FC Results

**Figure 2:**
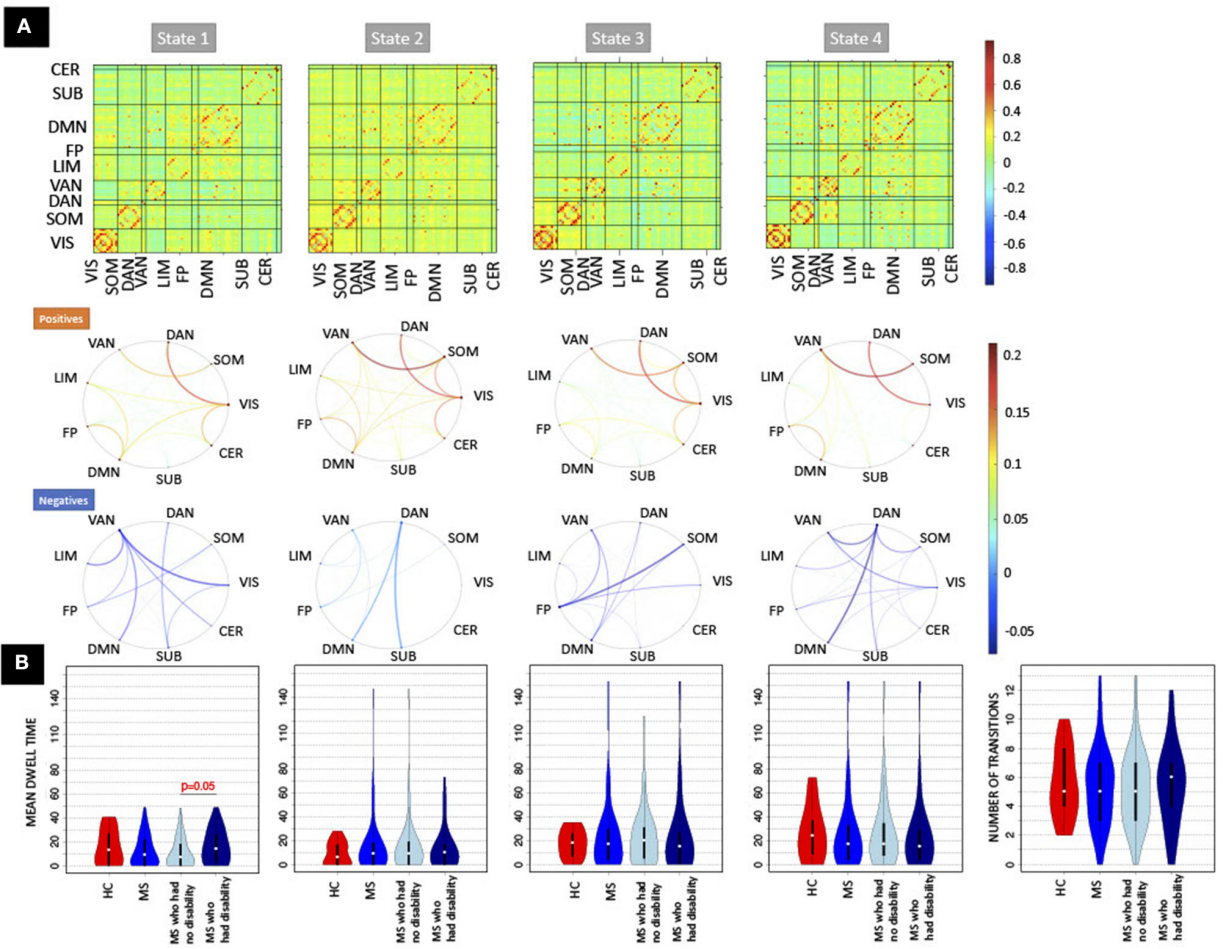
The recurring dynamic FC brain states obtained with k-means clustering and the dFC metrics (dwell time and number of transitions). **(A)** (Top row) Heat maps show the dynamic functional connectivity centroids for the four states, while [bottom two rows of **(A)**] the circle plots summarize the dFC centroids at a network level with the positive and negative dFC entries averaged separately. The color bar of the circle plot is not symmetrical, as to better visualize the network-level values. **(B)** The dFC metrics (mean dwell time and number of transitions) obtained for HC, pwMS, pwMS who had no disability, and pwMS who had evidence of disability. DAN, Dorsal Attention; VAN, Ventral Attention; LIM, Limbic; FP, Fronto-Parietal; DMN, Default-Mode Network; SUB, Subcortex; CER, Cerebellum; VIS, Visual; SOM, Somatomotor.

Four clusters in the dynamic FC analysis was identified as optimal (see Supplementary Figure 1). Figure 2 illustrates the 4 cluster centroids, or dynamic brain states (top panel), which are also summarized in the bottom panel by averaging the pairwise dFC values over Yeo network assignments separately for both the positive and negative values. All states show strong positive connections between ventral attention and somatomotor and between dorsal attention and visual networks, while the negative values vary more widely across the states. State 1 has more negative connections from ventral attention to visual and limbic networks. State 2 shows overall smaller magnitude negative connections and larger magnitude positive connections compared to other states. State 3 has more negative connections from fronto-parietal to somatomotor networks. State 4 differs from other states with larger magnitude negative connections from dorsal attention to default mode and ventral attention networks. Figure 2 also depicts the mean dwell time and total number of state transitions for HC, pwMS, pwMS who had no disability, and pwMS who had evidence of disability. A Student's *t*-test was used to compare the dFC metrics between groups. The pwMS who had disability had significantly higher dwell time compared to those without disability in State 1 (*p* = 0.05). While there was no significant difference in mean dwell time between groups in other states or in number of state transitions (*p* >0.05 for both comparisons), the pwMS with disability tend to have greater number of state transitions compared to HC and pwMS who had no disability. The transition probability between states are presented in Supplementary Figure 2.

### 3.3. Mass Univariate Group Comparison of Connectivity Measures

There were no significant differences in pairwise or regional FC and dFC between HC vs. pwMS, however, 24 pairwise SCs and 2 regions' SC node strengths (left and right accumbens) were significantly different between HC vs. pwMS after multiple comparison correction, see Supplementary Figure 3. There were no significant differences in pairwise and regional SC or FC between pwMS who had no disability vs. had evidence of disability (corrected *p* > 0.05 for all comparisons). However, the regional dFC in the right superior parietal was greater in pwMS who had evidence of disability compared to those without disability (corrected *p* = 0.02; see Supplementary Figure 4). PwMS with evidence of disability spent significantly more time in dFC brain state 1 compared to those pwMS with no disability (corrected *p* = 0.05), transition probability from state 4 to 3 trended toward greater values in HC compared to pwMS (uncorrected *p* = 0.03), and transition probability from state 3 to 2 trended toward greater values in pwMS who had evidence of disability compared to those with no disability (uncorrected *p* = 0.01). There was no significant difference or trend in number of transitions between HC vs. MS as well as between subgroups of pwMS.

### 3.4. Classification Results

**Figure 3:**
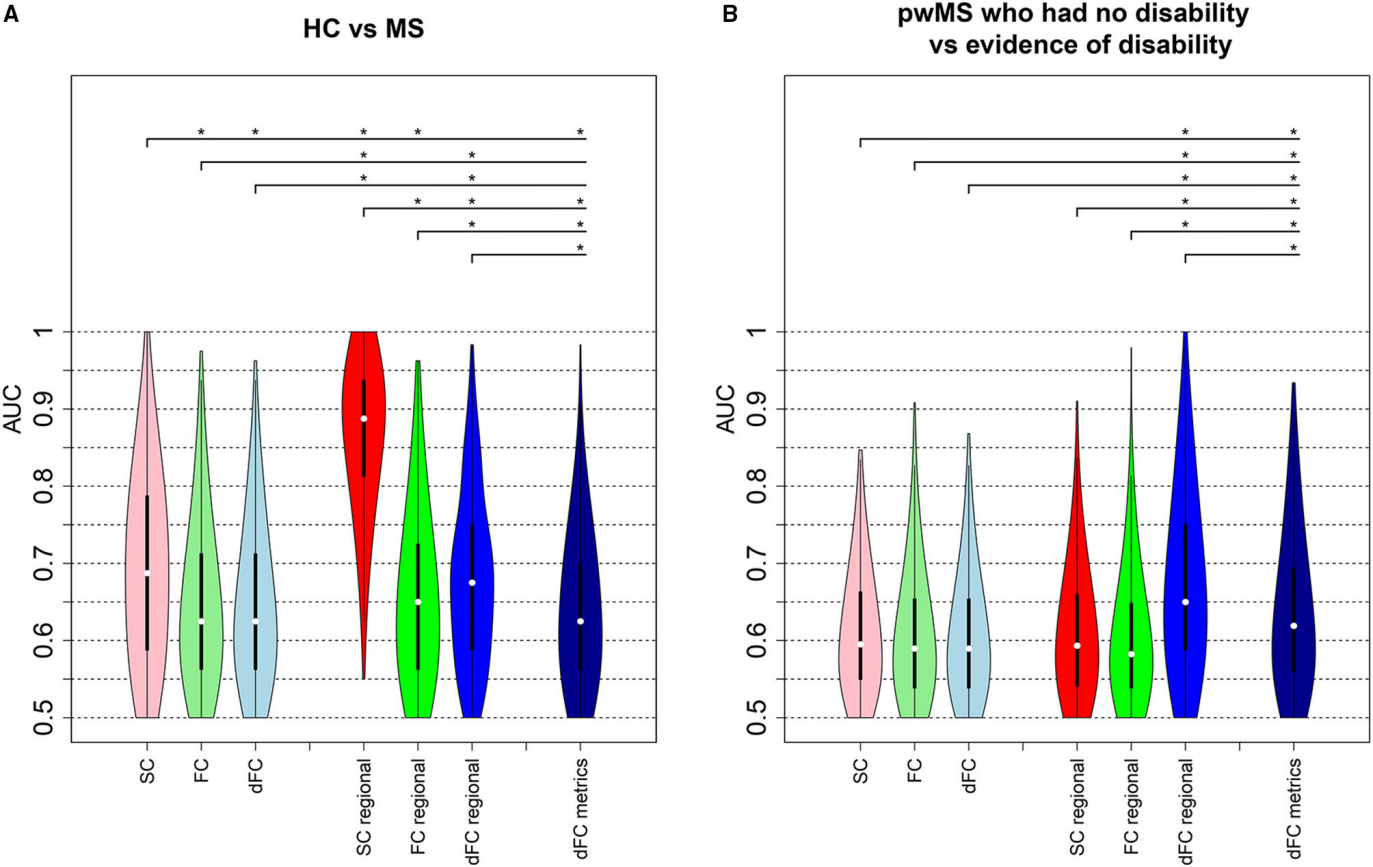
Classification analysis results. AUC values obtained from the models classifying **(A)** HC vs. pwMS and **(B)** pwMS according to their disability level. The first three violin plots show the AUC results from the models including pairwise connectivity information. The number of the imaging variables was 3,655 for pairwise SC and FC, while 14,620 (= 3655 × 4 dFC states) variables included in the pairwise dFC model. The next set of three violin plots show the AUC values from the regional models where regional SC and FC models each included 86 imaging variables and the regional dFC model included 344 (= 86 × 4 dFC states) imaging variables. The last model that included dFC metrics contained 21 imaging variables. The plots show the median (white dot), 1st and 3rd quartiles (black bar) over the 500 hold-out test sets. Asterisks indicate significant differences (*p* <0.05, BH corrected) in the distribution of AUC values between different models.

Figure 3 shows the distribution of AUC values (over the 500 hold-out test sets) for the models based on pairwise or regional SC, FC, and dFC separately as well as the model including dFC metrics for both classification tasks (HC vs. pwMS and pwMS disability subgroups). Unsurprisingly, the AUC results were generally higher for HC vs. pwMS classification than the AUCs obtained for the pwMS subgroup classification. For the HC vs. pwMS classification, the regional SC model performed significantly better than all other models, with a median AUC of 0.89. The regional models better classified HC and pwMS than pairwise models. The regional dFC model (node strength of the individual dFC cluster centroids) showed better classification accuracy than pairwise FC and pairwise dFC cluster centroids. For the classification of pwMS according to their disability level, the median AUC values ranged between 0.59 and 0.65, where the models that included regional dFC and dFC metrics performed significantly better than other models.

Supplementary Figure 6 shows other performance metrics (sensitivity, specificity, balanced accuracy, and F1) of all the models in classifying HC vs. pwMS and pwMS by disability level, respectively. Similar to AUC results, pairwise and regional SC models have better performance than other models in classifying HC vs. MS, while regional dFC and dFC metrics have better performance in distinguishing between pwMS having no disability vs. evidence of disability.

### 3.5. Feature Weights

**Figure 4:**
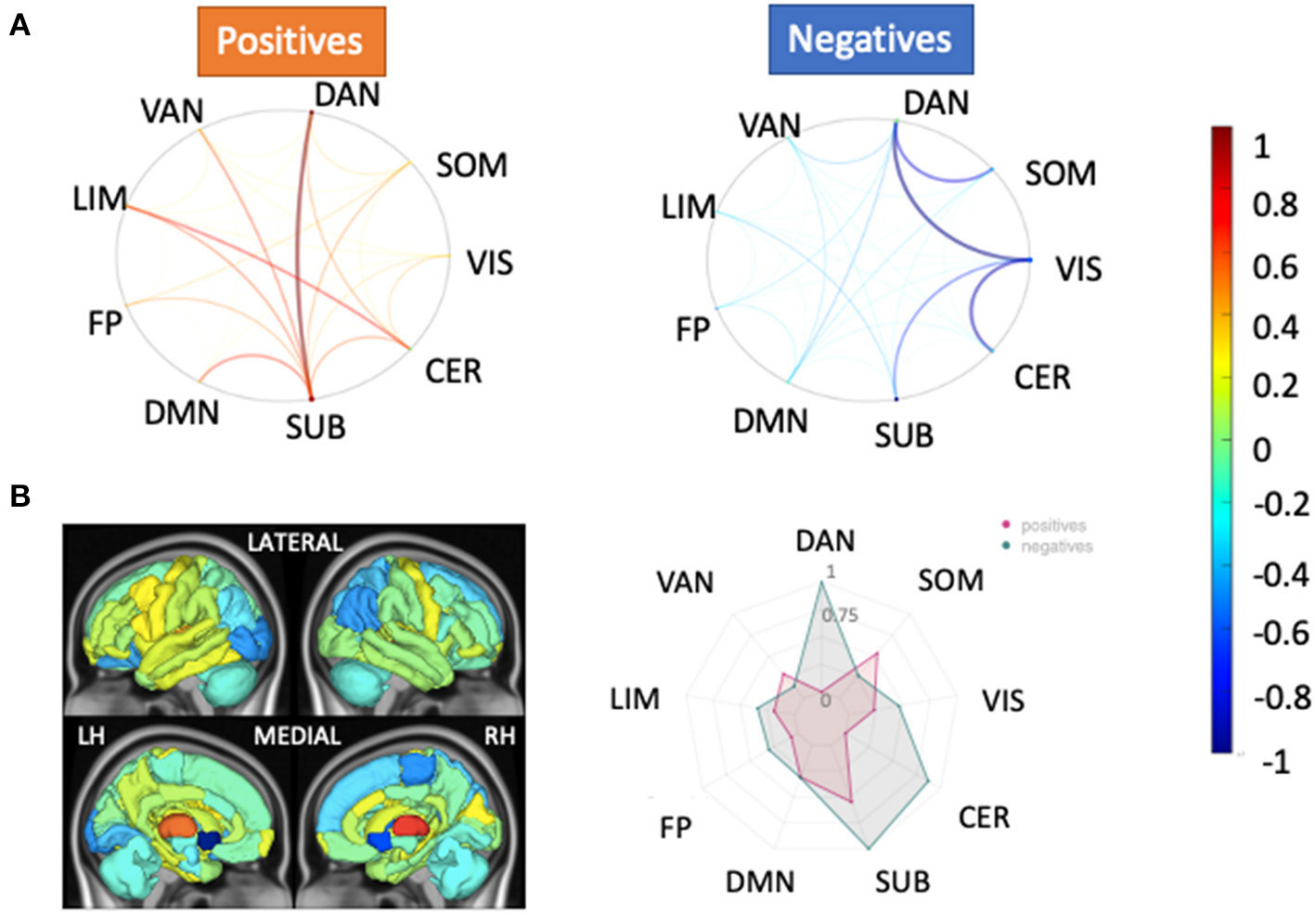
Relative feature weights of the SC models for the HC vs. pwMS classification task. The relative feature weights (scaled by the maximum magnitude feature weight) for the variables used in the two models with the best classification performance in the HC vs. pwMS task: **(A)** pairwise SC and **(B)** regional SC (node strength). The circle plots in **(A)** illustrate the positive (hot colors) and negative (cool colors) model feature weights, respectively, for the pairwise SC model, averaged across the Yeo functional networks. The glass brain and radial plot figures in **(B)** show the relative feature weights from the regional SC (node strength) model, where the redial plot shows the positive and negative values averaged over the Yeo functional networks. Negative values (cooler colors) indicate those connections where larger values were associated with greater probability of being in the HC group while positive values (hotter colors) indicate those connections where larger values were associated with greater probability of being in the pwMS group. DAN, dorsal attention; VAN, ventral attention; LIM, limbic; FP, fronto-parietal; DMN, default-mode network; SUB, subcortex; CER, cerebellum; VIS, visual; SOM, somato-motor.

Figure 4 depicts the scaled feature weights (relative to the maximum magnitude feature weight) for the pairwise and regional SC models that had the highest AUCs for the HC vs. pwMS classification task. Weaker SC between visual and dorsal attention/cerebellar networks and between somatomotor and dorsal attention networks, and stronger SC between the dorsal attention and subcortical networks were associated with being in the group of pwMS. This largely agreed with the regional SC feature weights showing weaker SC in regions of the dorsal attention, subcortical and cerebellar networks were associated with being in the group of pwMS.

**Figure 5:**
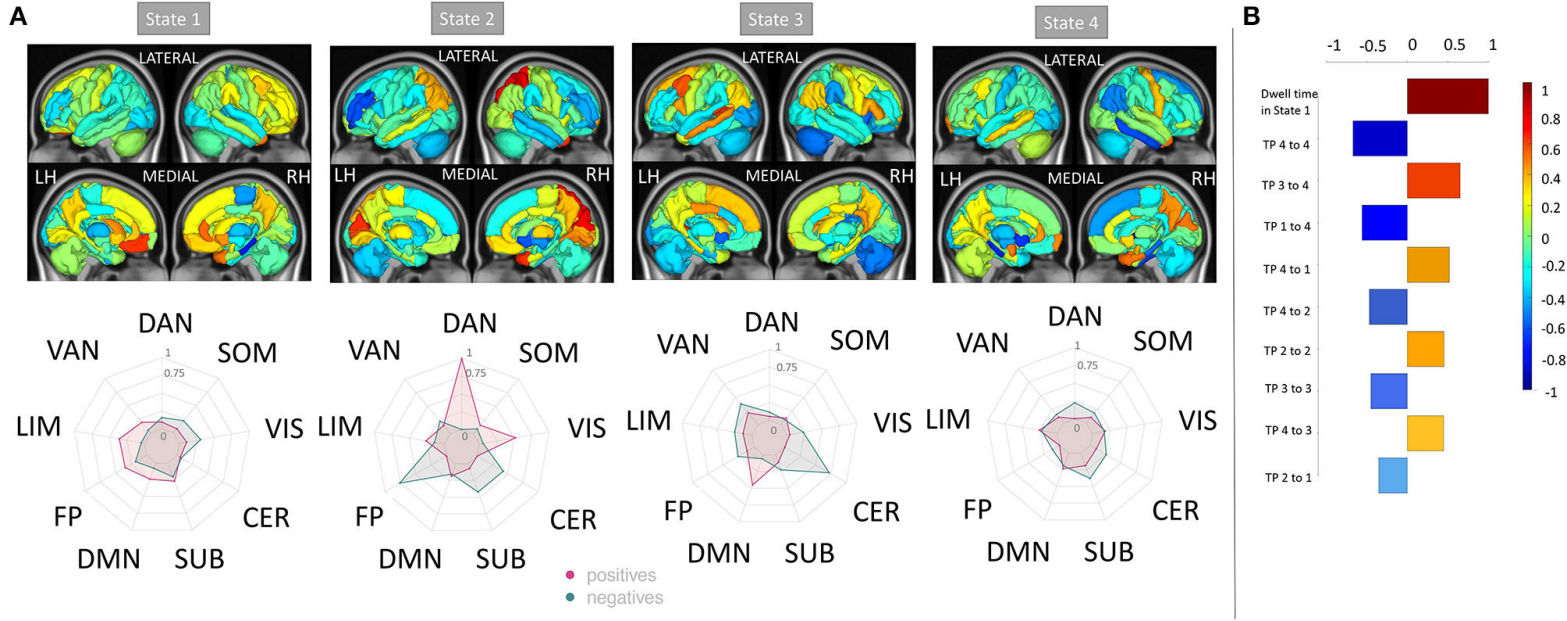
Relative feature weights of the regional dFC model for the MS subgroup classification task. The relative feature weights (scaled by the feature weight with maximum magnitude) of the variables used in classifying pwMS by disability status using **(A)** regional dFC (node strength) and **(B)** dFC summary metrics (top 10 important features). The glass brain and radial plots in **(A)** show the relative feature weights from the regional dFC model and summarize the average of the regional dFC values over the Yeo functional networks (positive and negatives averaged separately). Negative values (cooler colors) indicate those connections where larger values were associated with greater probability of being in the HC group while positive values (hotter colors) indicate those connections where larger values were associated with greater probability of being in the pwMS group. DAN, dorsal attention; VAN, ventral attention; LIM, limbic; FP, fronto-parietal; DMN, default-mode network; SUB, subcortex; CER, cerebellum; VIS, visual; SOM, somato-motor.

The regional dFC (node strength) model that had the best performance in classifying the pwMS into subgroups showed that increased dFC in the dorsal attention and visual networks of state 2, increased dFC in the default mode network of state 3, decreased dFC in the frontoparietal of state 2 and decreased dFC in the cerebellum of state 3 were most strongly associated with evidence of disability (see Figure 5). The univariate results were in concordance with these results and increased dFC in dorsal attention and visual networks of state 2 was found in pwMS who had evidence of disability compared to those without disability (see Supplementary Figure 4). In the dFC metrics model, dwell time in state 1, which was characterized by larger magnitude negative FC from the ventral attention network to several other networks, was the most important feature. This agreed with the univariate group comparisons indicating a significant increase in state 1 dwell time for pwMS with evidence of disability.

## 4. Discussion

In this study, we investigated the prediction ability of pairwise or regional SC, FC and dFC as well as dFC metrics in classifying HC vs. pwMS and pwMS who had no disability vs. evidence of disability. Our main findings were that (1) the regional SC (node strength) model had the highest AUC when discriminating between HC and pwMS, but the regional dFC and dFC metrics better distinguished pwMS into groups defined by disability level, (2) the most important regional SCs in distinguishing HC from pwMS were found in the dorsal attention, subcortical, and cerebellar networks, while regional dFC in the dorsal attention and visual networks were the most important in classifying pwMS into disability groups, and (3) mean dwell time in a state characterized by more negative FC from ventral attention to several other networks was the most important dFC metric for the classification of pwMS into disability groups.

### 4.1. Comparison With Previous Studies Using SC, Static FC, and Dynamic FC in MS

Previous studies have used statistical methods to differentiate between pwMS and HC, and pwMS according to their disability severity or phenotype (Richiardi et al., 2012; Leonardi et al., 2013; Stamile et al., 2015; Kocevar et al., 2016; Muthuraman et al., 2016; Ion-Mărgineanu et al., 2017; Zhao et al., 2017; Zhong et al., 2017; Saccà et al., 2018; Zurita et al., 2018) showed 82% sensitivity, similar to our results (median sensitivity = 0.85), in distinguishing pwMS from HC using static FC and/or lesion load. In one of the most similar studies to date in sample size, availability of multi-modal data types and nature of classification tasks, Zurita et al. (2018), showed that using SC and static FC resulted in high accuracy of 87% in classifying HC and pwMS but the accuracy of classifying pwMS according to EDSS dropped to 63%. Here, we show 77% balanced accuracy (AUC of 0.89) in pwMS vs. HC and 60% balanced accuracy (AUC = 0.64) in classifying pwMS by disability status. In contrast to our results, they found that static FC was more important than SC in classifying HC and pwMS. However, they did not investigate dynamic FC and their dMRI acquisition only had 15 directions compared to our higher resolution 55 directions acquisition, which likely means our SC matrices had increased sensitivity to detecting MS-related damage.

Ours is the first study to use dynamic FC to classify HC vs. pwMS or pwMS by disability level using machine learning. However, previous studies have investigated dFC differences between HC vs. pwMS as well as associations with cognition. One study showed that 50 CIS patients (47 of which converted to MS) had similar dFC properties compared to controls at baseline but one of the dFC measurements, the distance traveled in dynamic state-space, increased in CIS/pwMS over 2 years to levels above and beyond HC (Rocca et al., 2019). In another recent study, dFC metrics were compared between (i) pwMS and HC and (ii) pwMS with cognitive disability vs. preservation (d'Ambrosio et al., 2019). There, they showed no differences between HC and pwMS but pwMS without cognitive disability showed increased dynamic fluidity compared to pwMS with cognitive disability by exhibiting longer distance traveled in dynamic state-space, more dynamic states visited, and more frequent changes between states. A few limitations of that study were that the data was collected across 7 sites; the authors discuss this as having a non-negligible effect on the results. Still, both of these studies indicate that, at least early on in the disease, pwMS may compensate for MS-related damage by increased dynamism of FC.

### 4.2. Structural Damage to the Dorsal Attention Network Is Central in Distinguishing HC vs. pwMS

It has been suggested (Tian et al., 2021) that the parameter coefficients of the prediction models can be unreliable to assess the feature importance. Therefore, similar to our recent work that compared the prediction ability of observed vs. estimated SC and FC networks in classifying pwMS by disability status (Tozlu et al., 2021), here we report the important features that had high feature weight from the classification models and that also showed a larger difference in the mass univariate group comparisons.

Our study showed that the most discriminative pairwise SCs in distinguishing HC from pwMS were found from dorsal attention to subcortical and visual networks. SC node strength in regions in the dorsal attention network were also found to be important features in the HC vs. pwMS classification, as this network had the highest feature weight in the HC vs. pwMS classification model and the univariate analysis showed a large difference in this network between HC vs. MS. Connections between dorsal attention and other networks (limbic and frontoparietal) also had greater feature weights compared to other connections in the pairwise FC model. The prominence of the dorsal attention network in both analyses (classification and univariate analyses) is in line with a previous study that compared dFC metrics between HC and pwMS and found decreased dFC within dorsal attention in pwMS compared to HCs (Huang et al., 2019).

### 4.3. Dynamic FC Metrics May Capture Compensatory Functional Upregulation in pwMS

In our study, the univariate analyses showed decreased pairwise structural connections between dorsal attention and visual networks in pwMS with evidence of disability. The univariate analysis as well as the feature weights from the classification models showed that increased dFC in the dorsal attention network was associated with evidence of disability in MS. Moreover, the right superior parietal region of the dorsal attention network was the only region which was significantly higher in the pwMS who had evidence of disability compared to those without disability. We hypothesize the dorsal attention network's increased dFC in pwMS who had evidence of disability could be the result of either a pathological or compensatory upregulation of functional coordination, in response to disease-related damage to SC, i.e., the “less wiring more firing” phenomena (Daselaar et al., 2015). This provides further evidence that MS is characterized by damage to the SC but disability level within pwMS may be more related to functional compensation, specifically the level of dynamism of FC that is reflected in the dFC measures.

### 4.4. Decreased Connectivity in the Cerebellum Is Related to Disability

Our recent study that investigated the association between structural disconnectivity due to paramagnetic rim lesions and disability in MS showed that the cerebellum is one of the most important regions for the classification of pwMS by disability status and, further, that greater damage to the cerebellum is related to worse disability in MS (Tozlu et al., 2020). Previous studies have also shown the association between motor/cognitive disability and altered FC in the cerebellum (Dogonowski et al., 2014; Pasqua et al., 2020). Our results were in concordance with these previous findings in that decreased connectivity in the cerebellum was associated with evidence of disability in pwMS in all regional models (SC and dFC of states 2 and 3).

### 4.5. Limitations

The main limitations of our study were the cross-sectional nature and size of the sample. We were restricted to inferring cross-sectional relationships of brain networks properties and disability; a more clinically applicable model would be one capable of predicting with reasonable accuracy future disability for better patient management. There were only 33 pwMS who had evidence of disability and 19 controls which limited the ability to train robust models accurate in novel data. Future work including larger, longitudinal datasets from a similar cohort are required to validate the findings of the current study. EDSS primarily captures physical disability, so this is likely what is being mapped in this work. Future studies using more specific measures of different types of disability including cognition may allow further insights about brain-behavior relationships. In addition, the MRI acquisition parameters could be improved to obtain higher resolution information, including reducing the TR of the fMRI scan, increasing the duration of the entire fMRI scan, and increasing the number of *b*-values in the dMRI scan. Finally, in our dFC analysis, the BOLD time series was divided using a fixed window length; however, wavelet transforms may allow different lengths for different frequency bands and will also be explored in future studies.

## 5. Conclusion

In conclusion, regional SC proved to be the most discriminative modality in classifying HC vs. pwMS, and pwMS exhibited weaker SC within the dorsal attention network, cerebellum, and subcortex. Furthermore, models including dFC metrics outperformed others in classifying pwMS into disability status categories; there, the most important regional dFCs were in the dorsal attention and visual networks and the most important dFC metric was dwell time in a state characterized by more negative FC from ventral attention to other networks. These results suggest that damage to SC are hallmarks of MS, while dynamic FC may reveal functional connectivity differences that are associated with varying levels of disability in pwMS. Various brain connectivity network approaches may enable more accurate prognoses and, possibly, a better understanding of disease mechanisms, eventually leading to the development of novel therapeutics.

## Data Availability Statement

The raw data supporting the conclusions of this article will be made available by the authors, without undue reservation.

## Ethics Statement

The studies involving human participants were reviewed and approved by Weill Cornell IRB. The patients/participants provided their written informed consent to participate in this study.

## Author Contributions

CT helped with image post-processing, carried out the statistical analyses, and wrote the article. KJ collected the data, performed pre- and post-processing of MRI data, and reviewed the article. SG collected the data, helped the interpret results, and reviewed the article. AK designed and supervised the study, collected the data, and edited the article. All authors contributed to the article and approved the submitted version.

## Funding

This work was supported by the NIH R21 NS104634-01 (AK), NIH R01 NS102646-01A1 (AK), and grant UL1 TR000456-06 (SG) from the Weill Cornell Clinical and Translational Science Center (CTSC).

## Conflict of Interest

The authors declare that the research was conducted in the absence of any commercial or financial relationships that could be construed as a potential conflict of interest.

## Publisher's Note

All claims expressed in this article are solely those of the authors and do not necessarily represent those of their affiliated organizations, or those of the publisher, the editors and the reviewers. Any product that may be evaluated in this article, or claim that may be made by its manufacturer, is not guaranteed or endorsed by the publisher.
