# Supplementary material for: Dynamic Functional Connectivity Better Predicts Disability Than Structural and Static Functional Connectivity in People With Multiple Sclerosis
Source: Front Neurosci. 2021 Dec 13;15:763966. doi: 10.3389/fnins.2021.763966 (PMC8710545; doi:10.3389/fnins.2021.763966)
Supplement: Supplementary file 1 [file Data_Sheet_1.PDF]

1 Supplementary Document

1.1 Dynamic functional connectivity analysis

1.1.1 Estimation of the cluster number

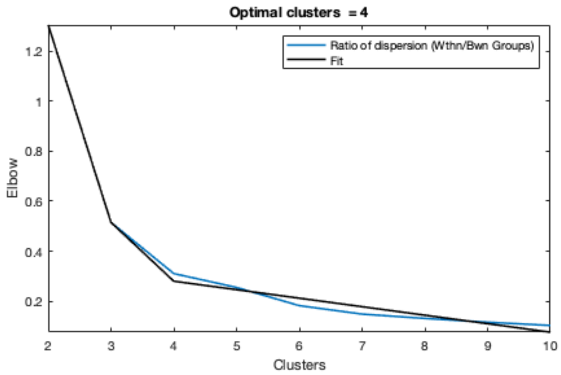

Figure 1: Finding the optimal state number. The selected optimal number of centroid states was 4, a number estimated using the elbow criterion, defined as the ratio of within-cluster to between-cluster distances.

1.1.2 Difference in transition probability

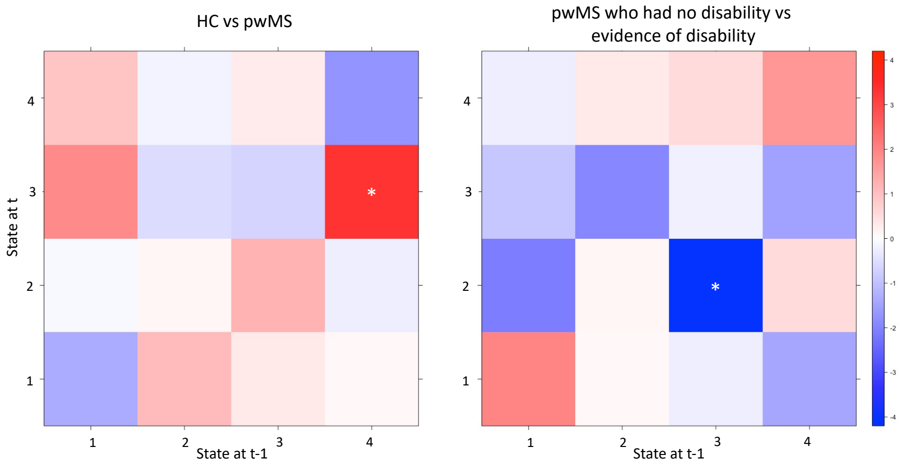

Figure 2: Differences in transition probabilities between two groups. Comparison of transition probabilities between dynamic states. Group differences are visualized via -log(p)*sign(difference in mean), where p here is the uncorrected p. The p-values were obtained using t-test. Differences were computed using the transition probabilities in pwMS - HC and in pwMS who had evidence of disability - no disability. * indicates that the uncorrected p-value is less than 0.05

1.2 Comparison of pairwise and regional connections between groups

1.2.1 Structural and functional connectivity

To minimize the effect of false positives in the tractography, only the entries in the SC that were nonzero in more than half of the controls were tested for group differences. There was no significant differences in pairwise or regional FC between HC vs pwMS, however, 24 pairwise SCs and 4 regional SC (node strength) were significantly different between HC vs pwMS after multiple comparison p-value corrections, see Figure 3. Unsurprisingly, the magnitude and number of the SC differences where HC $>$ pwMS were much larger than the magnitude and number of the differences where pwMS $>$ HC. The SC and FC from dorsal attention to various networks such as subcortex, limbic, somatomotor, and default mode networks were higher in pwMS compared to HC and in pwMS who had evidence of disability compared to pwMS who had no disability. The SC and FC between visual and cerebellum were also commonly found greater in HC compared to pwMS and in pwMS who had no disability compared to those who had evidence of disability. There was no significant difference in pairwise and regional SC and FC between pwMS who had no disability vs had evidence of disability (corrected p-value $>$ 0.05 for all comparisons).

The differences in regional SC between HC vs pwMS were much larger than other regional FC differences between HC vs pwMS as well as SC and FC between two disability groups in MS.

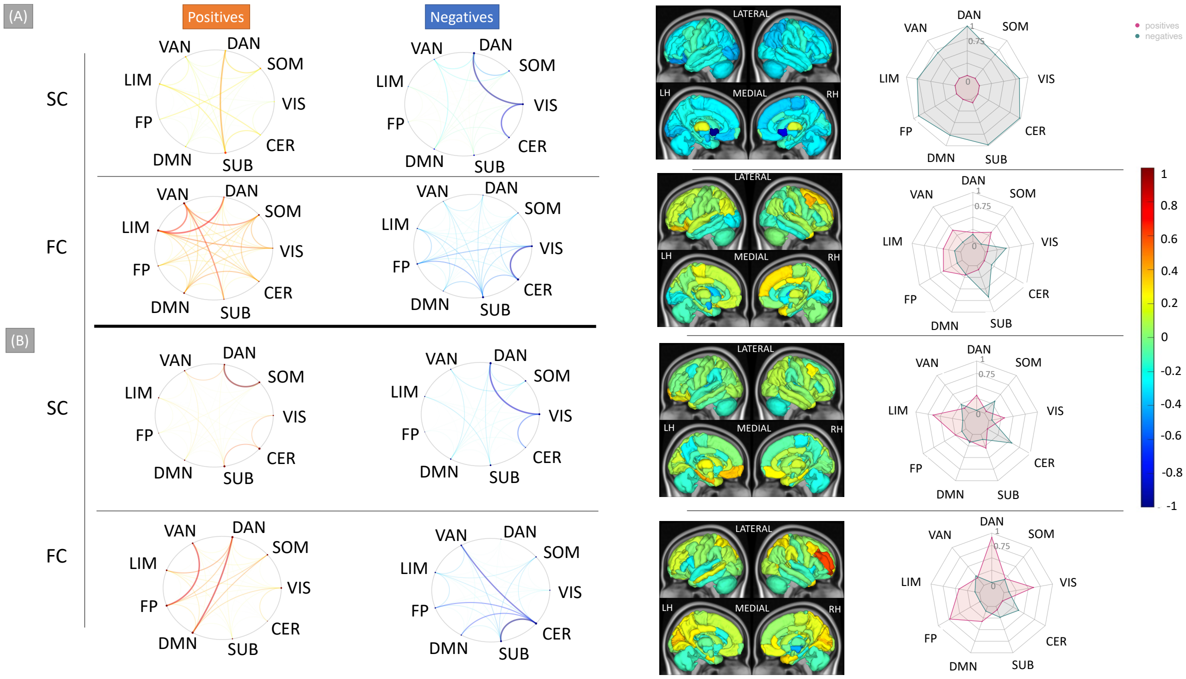

Figure 3: The difference in connections between (A) HC vs pwMS and (B) the no disability vs evidence of disability groups, where positive values (hotter colors) indicate pwMS had higher connectivity compared to HC (pwMS with evidence of disability had higher connectivity than those who had no disability), while negative values (cooler colors) indicate pwMS had weaker connectivity than HC (=pwMS with evidence of disability had weaker connectivity than those who had no disability). Group differences are visualized via -log(p)*sign(difference in mean or median), where p here is the uncorrected p. The p-values were obtained using t-test to compare FC, while Wilcoxon rank-sum test was performed to compare SC values between groups. Difference in mean was used for FC, while difference in median was computed for SC. The circle and radial plots summarize the differences by representing the mean of the positive and negative differences between functional network assignments (7 networks from the Yeo atlas, plus cerebellum and subcortex). The absolute value of the mean negative regional connections were presented in the radial plots. *Note: different colorbars used for the glass brain figures as the range in SC difference between HC vs MS was much larger than FC between HC vs pwMS and SC-FC between two disability groups in MS. DAN= Dorsal Attention, VAN= Ventral Attention, LIM= Limbic,FP= Fronto-Parietal, DMN= Default-Mode Network, SUB= Subcortex, CER= Cerebellum, VIS=Visual, and SOM= Somatomotor

1.2.2 Dynamic functional connectivity

Figure 4 shows the differences in pairwise dFC and regional dFC (node strength) between the two sets of groups. PwMS with evidence of disability had higher FC between dorsal attention and cerebellum, but lower FC between dorsal attention and default mode networks in State 1. All regional connections and the networks associated with these regions in State 1 were greater in pwMS who had evidence of disability than those who had no disability, in particular, frontoparietal showed the greatest difference between these two disability groups. Higher FC between dorsal attention and cerebellum in pwMS with evidence of disability as compared to those who had no disability was also found in State 2 as well as in HC as compared to pwMS in state 4. State 3 was characterized with greater regional dFC in HC compared to pwMS and in pwMS who had no disability than those with evidence of disability, where cerebellum and subcortex showed the greatest difference between HC and pwMS and ventral attention showed the greatest difference between two disability groups in State 3.

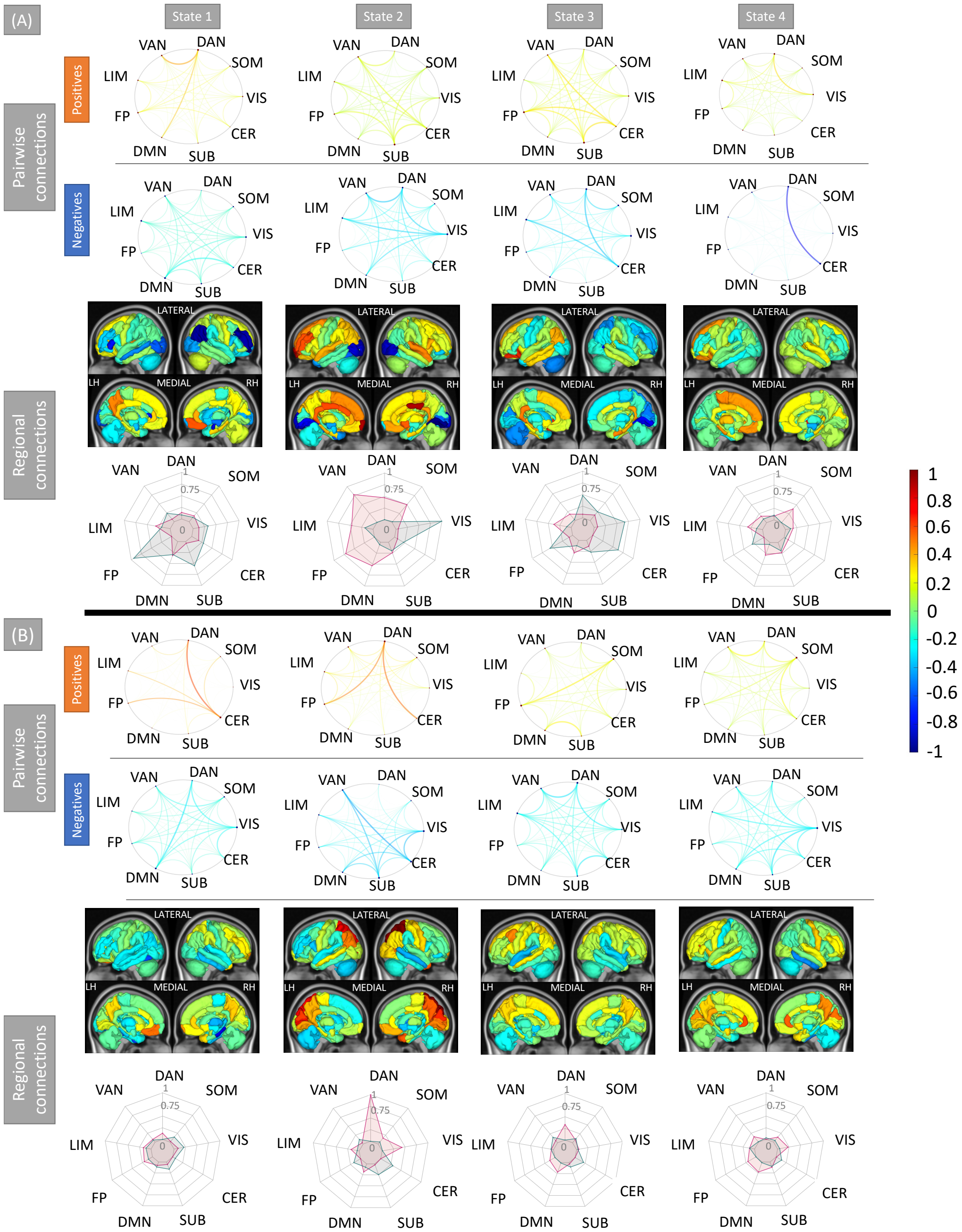
(A)
State 1
State 2
State 3
State 4
Pairwise connections
Positives
Negatives
Regional connections
VAN
DAN
SOM
VIS
CER
SUB
DMN
FP
LIM
LATERAL
MEDIAL
LH
RH
1
0.75
0
(B)
1
0.8
0.6
0.4
0.2
0
-0.2
-0.4
-0.6
-0.8
-1

Figure 4: The difference in pairwise and regional dFC between (A) HC vs pwMS and (B) pwMS who had no disability vs evidence of disability, where positive values (hotter colors) indicate pwMS had higher dFC than HC (=pwMS with evidence of disability had higher dFC than those with no disability), while negative values (cooler colors) indicate pwMS had weaker dFC than HC (=pwMS with evidence of disability had weaker dFC than those with no disability). Group differences are visualized via -log(p)*sign(difference in mean), where p here is the uncorrected p. The p-values were obtained using t-test to compare dFC between two groups. The radial plots summarize the differences of dFC by representing the mean of the positive and negative differences in the same functional networks used for the circle plot. The absolute value of the mean negative connections were presented in the radial plots. DAN= Dorsal Attention, VAN= Ventral Attention, LIM= Limbic,FP= Fronto-Parietal, DMN= Default-Mode Network, SUB= Subcortex, CER= Cerebellum, VIS=Visual, and SOM= Somatomotor

1.3 Classification analysis

1.3.1 Linear regression with ridge regularization

Linear regression is one of the most widely used statistical methods available today. However, over-fitting problem may occur when performing linear regression with an high dimensional data. Ridge regression estimates the parameter coefficient ($\hat{\beta}_{ridge}$) by applying L_2 penalty as follows:

$$\hat{\beta}_{ridge} = (X^T X + \lambda I)^{-1} X^T Y \qquad (1)$$

where X is the input and Y is the output.

$\lambda \geq 0$ is a tuning parameter for the penalty, which is determined separately. Best λ that maximizes the classification accuracy (AUC) in the inner loop was chosen as the best hyperparameter to fit the model in the outer loop. In each inner loop, the best value of λ was searched in $[10^{-3}, 10^{+3}]$ with steps of 10.

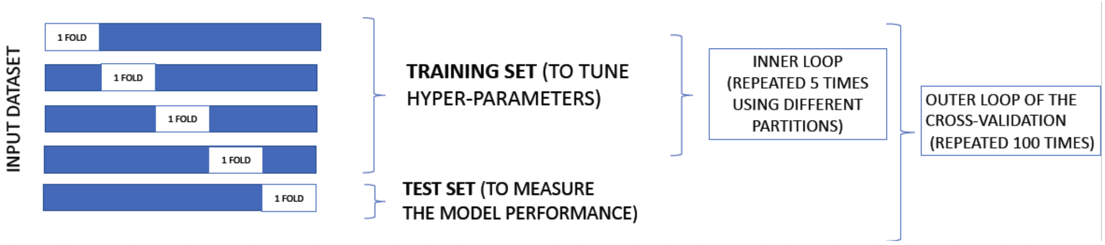

Figure 5: The nested cross-validation approach used in this study.

The important variables were identified using both univariate statistics (t-test or Wilcoxon rank sum test based on the connectivity type) and the mean feature weights (the beta parameter coefficients) over all 500 models (100 partitions of the data into 5 folds) (1). Regional and region-pair feature weights were also summarized at a functional network level by assigning each of the 68 cortical regions to one of seven canonical functional networks (2); subcortex and cerebellum were also added as networks.

1.3.2 Results of the classification analysis

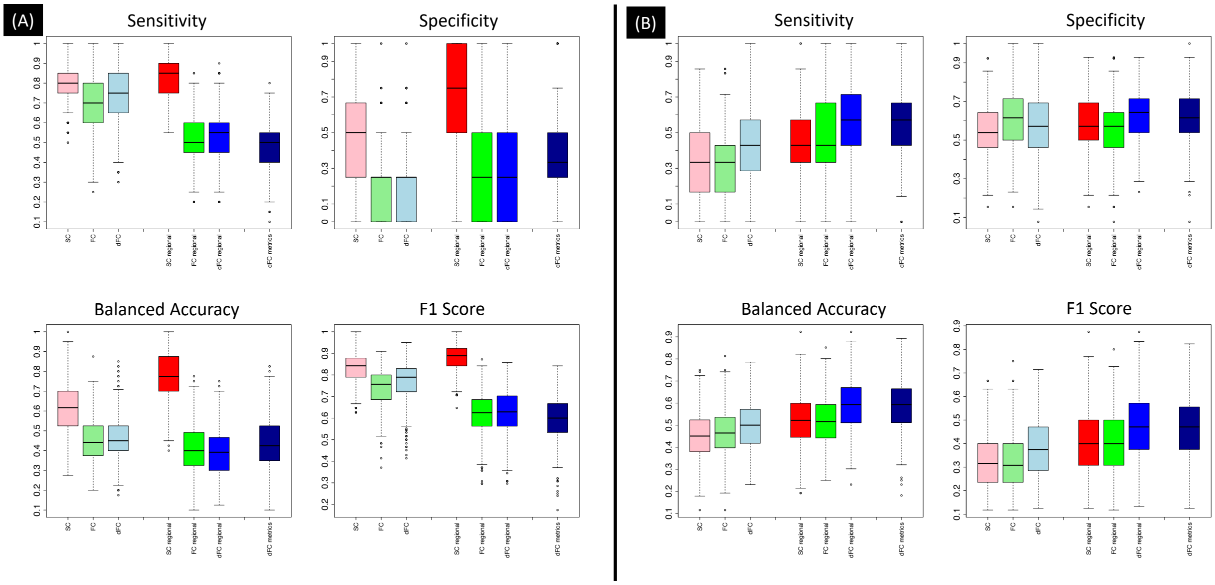

Figure 6: The classification performance the models. Sensitivity, specificity, balanced accuracy, and F1 score computed by each model in classifying (A) HC vs pwMS and (B) pwMS by disability status.

1.3.3 Feature weights computed with the classification models

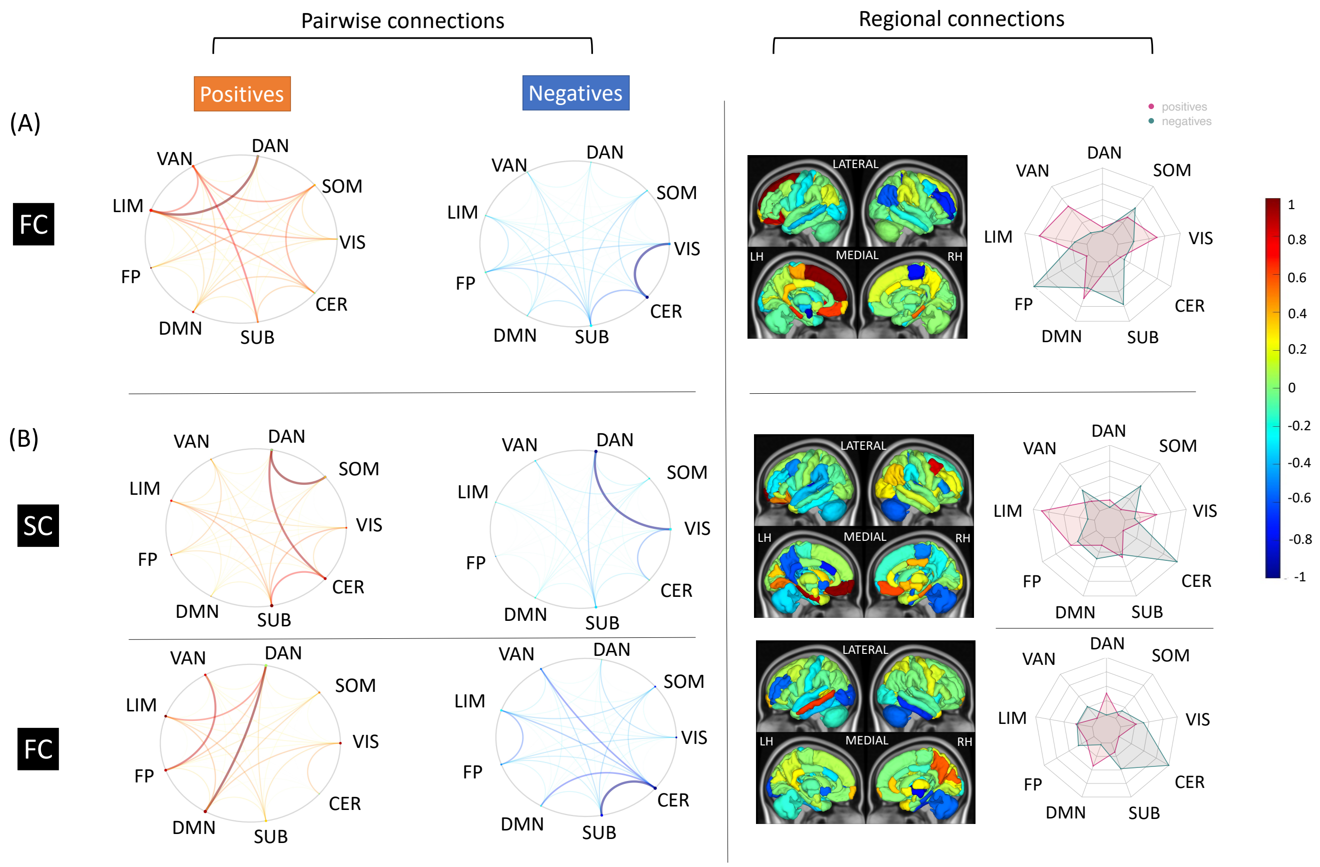

Figure 7: The relative feature weights of the structural and functional connections in classifying (A) HC vs pwMS and (B) pwMS by disability status. The circle plots represent the positive and negative values of the feature weights from the pairwise models. The positive parameter coefficients were indicated with hotter colors, while negative parameter coefficients were indicated with cooler colors. The glass brain and radial plot figures show the feature weights from the regional models. The relative feature weights were computed by dividing the parameter coefficients with the maximum value of the absolute parameter coefficients. The circle and radial plots presented using 9 networks (7 networks from Yeo 7 atlas, the cerebellum, and subcortex) that included 86 regions from FreeSurfer atlas. The radial plots summarize the relative feature weights by representing the mean of the positive and negative weights in the same functional networks used for the circle plot. DAN= Dorsal Attention, VAN= Ventral Attention, LIM= Limbic,FP= Fronto-Parietal, DMN= Default-Mode Network, SUB= Subcortex, CER= Cerebellum, VIS=Visual, and SOM= Somatomotor

1

References

[1] Y. Tian, A. Zalesky, D. Ye, T. Level, A. G. Building, A. Z. Level, and A. Gilbert Building, “Machine learning prediction of cognition from functional connectivity: Are feature weights reliable?,” *bioRxiv*, p. 2021.05.27.446059, 5 2021.

[2] B. T. T. Yeo, F. M. Krienen, J. Sepulcre, M. R. Sabuncu, D. Lashkari, M. Hollinshead, J. L. Roffman, J. W. Smoller, L. Zöllei, J. R. Polimeni, B. Fischl, H. Liu, and R. L. Buckner, “The organization of the human cerebral cortex estimated by intrinsic functional connectivity.,” *Journal of neurophysiology*, vol. 106, pp. 1125–65, 9 2011.
